# Association between depressive symptoms, use of antidepressant medication and the metabolic syndrome: the Maine-Syracuse Study

**DOI:** 10.1186/s12889-016-3170-2

**Published:** 2016-06-10

**Authors:** Georgina E. Crichton, Merrill F. Elias, Michael A. Robbins

**Affiliations:** Alliance for Research in Exercise, Nutrition and Activity (ARENA), Sansom Institute for Health Research, University of South Australia, GPO Box 2471, Adelaide, South Australia 5001 Australia; Department of Psychology, University of Maine, Orono, ME USA; Graduate School of Biomedical Sciences and Engineering, University of Maine, Orono, ME USA

**Keywords:** Depressed mood, Antidepressants, Metabolic syndrome

## Abstract

**Background:**

Both depression and the metabolic syndrome (MetS) are two major public health issues. The aim of this study was to examine associations between depressive symptoms, the use of antidepressant medications, and the prevalence of MetS.

**Methods:**

Cross-sectional analyses were undertaken on 970 participants from the Maine-Syracuse Study. Depressive symptoms were measured using two self-reported depression scales, the Center for Epidemiological Studies Depression Scale (CES-D), and the Zung self-rating depression scale. Antidepressant medication use was also self-reported. MetS was defined according to the recent harmonized criteria.

**Results:**

The risk of MetS were approximately 79 and 86 % higher for those in the highest quartile for the CESD and the Zung (CES-D: OR = 1.79, *p* = 0.003; Zung: OR = 1.71, *p* = 0.006), compared to those in the lowest quartile. With adjustment for socio-demographic variables, lifestyle factors and C-reactive protein (CRP), risk was attenuated, but remained statistically significant for the CES-D. In those who reported using antidepressant medication, the odds of having MetS were over 2-fold higher (OR = 2.22, *p* < 0.001, fully adjusted model), compared to those who did not use antidepressants. Both measures of depressed mood were also associated with low high density-lipoprotein (HDL) cholesterol levels. Antidepressant use was associated with elevated fasting plasma glucose concentrations, hypertension, and low HDL-cholesterol.

**Conclusion:**

Depressive symptoms and the use of antidepressant medications are associated with the prevalence of MetS, and with some of the individual components of the syndrome.

## Background

The clustering together of abdominal obesity, hypertension, dyslipidemia and insulin resistance is referred to as the metabolic syndrome (MetS). A syndrome can be regarded as “a clustering of factors that occur together more often than by chance alone and for which the cause is often uncertain” ([1], p.1641). The worldwide prevalence of MetS is increasing, in part attributable to the rise in obesity [1]. While there is not a single known cause for MetS, a number of non-modifiable factors including age, genetics, ethnicity and sex, influence the syndrome’s prevalence [2]. Having MetS is associated with a 1.7-fold increase in the risk for cardiovascular disease (CVD) [3]. For non-diabetics with MetS, the likelihood of developing future type 2 diabetes increases seven-fold [4], further augmenting CVD risk. The presence of MetS may also be a future predictor of mortality [5, 6]. Research is needed to generate public health measures to combat MetS and develop interventions for at-risk individuals.

Like MetS, depression is associated with an increased risk of diabetes [7] and CVD [8]. Depression is one of the most common psychological illnesses affecting adults in the United States (US), with over 15 million adults having had at least one major depressive episode in 2013 [9]. With both MetS and depression being recognised as major public health challenges, attention has been drawn to associations between the two.

A recent systematic review and meta-analysis investigated the current evidence for an association between depression and MetS from 29 cross-sectional and 11 prospective studies [10], and concluded that a bidirectional association exists. While a growing amount of literature suggests that depressive symptoms and more severe depressive episodes are associated with MetS [10–13], some inconsistent results have been reported [14, 15], and the direction of this association remains uncertain.

Fewer studies have examined any association between antidepressant use and MetS. A recent study suggests that antidepressants are a stronger predictor of type 2 diabetes than are depressive symptoms [16]. As antidepressants may directly effect components of MetS, in particular, weight gain [17–19], it is important to consider antidepressant use in relation to the syndrome. The PPP-Botnia Study (PPP = prevalence, prediction, and prevention of diabetes), a large Finnish population-based study, recently examined associations between depressive symptoms, use of antidepressant medication and MetS [20]. In this study the odds for having MetS increased over 10 % for each standard deviation increase in depressive symptoms, and users of antidepressant medication had more than 50 % increased odds for having MetS. More studies such as this one are needed to extend what is currently known about these relations. Few, if any, studies have used more than a single depression scale, and it is important to know if similar results are obtained for two different mesures of depressed mood.

Therefore, the present study also reports associations between depressive symptoms using two different self-report scales one prominent in the epidemiology literature (Zung) and one prominent in the psychology literature (CES-D), antidepressant use, and MetS prevalence, and also tests relations with the individual components of MetS, in a US community-based sample. Further, we examine if any potential associations vary according to apolipoprotein ε4 (APOE ε4) status. It is hypothesized that there will be a positive association between self-reported depressive symptoms and MetS prevalence, and that those on antidepressant medication will have a higher likelihood of having MetS.

## Methods

### Participants

Data were obtained from participants of the Maine-Syracuse Longitudinal Study (MSLS), which commenced in 1975 and was designed to measure blood pressure and cognitive performance [21, 22] and expanded at Wave 6 to examine other cardiovascular risk factors. The MSLS employs a time-lagged-longitudinal-cohort design with new subjects featuring an initial wave of data collection and then new subjects recruited into the study every five years with the same recruitment procedures as employed for every wave.

Sample characteristics at Wave 6 (Table 1) differed in two ways from those at the initial wave of testing, approximately 25 years earlier. Subjects were older (Wave 1 mean age = 48.0 ± 16.0 years), there were fewer women (Wave 1 proportion = 56 %), and mean blood pressures were lower due to the need to treat subjects once hypertension was detected (Wave 1: mean systolic = 137 ± 28 mmHg, mean diastolic = 82 ± 18 mmHg). In this context, it is important to note that our analysis was cross-sectional at Wave 6 and our goal was not to duplicate sample charcteristics from a study begun years ealier. No comparison between our MetS data or our covariates at Wave 6 with Wave 1 are possible because data pertienet to MetS were all collected for the first time at Wave 6.

**Table 1:** Demographic, cardiovascular and health variables for Maine-Syracuse Longitudinal Study (*N* = 970) participants according to metabolic syndrome status

	MetS				
Variable	No		Yes		*P*-value
	*n* = 539		*n* = 431		
	M or %	SD	M or %	SD	
Age, years	60.7	13.4	63.7	11.9	<0.001
Gender					0.1
Males	38.8		43.9		
Females	61.2		56.1		
Education, years	15.1	2.6	14.1	2.7	<0.001
Race					0.019
Caucasian	94.2		90.3		
African American	5.8		9.7		
Smoking, no cigarettes/day	1.0	4.2	1.8	6.4	0.027
BMI, kg/m^2^	27.1	4.5	32.1	6.2	<0.001
Physical activity, MET mins/day^a^	1517	1803	880	1370	<0.001
Total cholesterol, mg/dL	204.8	37.1	197.0	42.2	0.002
HDL cholesterol, mg/dL	59.2	5.2	46.7	12.4	<0.001
LDL cholesterol, mg/dL	125.8	32.0	114.5	33.8	<0.001
Systolic BP, mmHg	124.8	21.0	138.5	19.9	<0.001
Diastolic BP, mmHg	68.6	9.9	72.8	9.7	<0.001
Fasting blood glucose, mg/dL	90.6	15.4	109.5	35.9	<0.001
Waist circumference, cm	88.8	12.4	103.6	14.0	<0.001
CRP, mg/L	0.36	0.43	0.52	0.53	<0.001
Triglycerides, mg/dL	101.9	47.0	192.4	143.3	<0.001
Alcohol intake, g/week	41.6	74.6	29.2	62.3	0.006
Depressed mood, CES-D score^b^	6.8	6.4	8.5	7.3	<0.001
Depressed mood, Zung score^b^	41.1	8.5	43.8	10.1	<0.001
% using antidepressants	8.3		15.1		0.001
% Obese (BMI ≥ 30 kg/m^2^)	20.8		61.4		<0.001
% Diabetes mellitus^c^	2.0		25.5		<0.001
% Hypertension^d^	40.8		87.5		<0.001
% CVD^e^	8.0		22.0		<0.001
% APOE ε4	26.9		27.0		0.9

*APOE ε4* apolipoprotein ε4, *BMI* body mass index, *BP* blood pressure, *CES-D* Center for Epidemiologic Studies Depression Scale, *CRP* C-reactive protein, *CVD* cardiovascular disease, *MetS* metabolic syndrome, *MET* metabolic equivalent, *MSLS* Maine-Syracuse Longitudinal Study

^a^Includes moderate and intense self-reported physical activity

^b^Higher scores indicates greater number of depressive symptoms

^c^Fasting plasma glucose ≥ 126 mg/dL or taking anti-diabetic medication

^d^Systolic BP ≥ 140 mmHg and/or diastolic BP ≥ 90 mmHg or taking anti-hypertensive medication

^e^Includes myocardial infarction, coronary artery disease, heart failure, angina pectoris, transient ischemic attack

The only recruitment exclusions (common to all waves) were institutionalization, diagnosed psychiatric disorder and alcoholism. Participants for the present study were those returning for the sixth (2001-2006) study wave. Details of initial study recruitment have been previously described [21–24]. Beginning with 1049 individuals, participants for the present study were excluded for the following reasons: missing cardiometabolic, depression or medication data (*n* = 36), acute stroke (*n* = 28), probable dementia (*n* = 8), undertaking dialysis treatment (*n* = 5), inability to read English (*n* = 1), and prior alcohol abuse (*n* = 1). The final sample with complete wave 6 data comprised 970 individuals, aged 23 to 98 years.

Stroke, defined as a focal neurological deficit of acute onset persisting more than 24 h, was based on self-report and was confirmed by a record review indicating a diagnosis of acute stroke. Clinical diagnoses of dementia were determined from cognitive data and medical records using the National Institute of Neurological and Communicative Diseases and Stroke/Alzheimer’s Disease and Related Disorders Association (NINCDS-ADRDA) criteria [25] and confirmed using the ICD-10 Guidelines [26].

### Ethics, consent and permissions

This study was conducted according to the guidelines established by the Declaration of Helsinki and all procedures were approved by the University of Maine Institutional Review Board. Written informed consent was obtained from all subjects.

## Procedure

### Metabolic syndrome

Metabolic syndrome was defined according to the International Diabetes Federation and the American Heart Association/National Heart, Lung, and Blood Institute, which requires 3 of 5 risk factors to be present [1] namely abdominal obesity (WHO waist circumference cut-offs for Caucasian population: men ≥94 cm, women ≥80 cm), elevated blood pressure (systolic: ≥130 and/or diastolic ≥85 mmHg), elevated fasting plasma glucose (≥5.6 mmol/L or ≥100 mg/dL), elevated triglycerides (≥1.7 mmol/L or ≥150 mg/dL), reduced high density lipoprotein (HDL) cholesterol (<1.0 mmol/L or <40 mg/dL for males; <1.3 mmol/L or <50 mg/dL for females).

Standard assay methods were employed [21] to obtain fasting plasma glucose (mg/dL), total cholesterol (TC, mg/dL), low-density lipoprotein cholesterol (LDL, mg/dL), high-density lipoprotein cholesterol (HDL, mg/dL), triglycerides (mg/dL) and C-reactive protein (CRP, mg/L), following an overnight fast. Waist circumference (in centimetres) was taken over light clothing, using a non-extendable measuring tape, at the level of the iliac crest. Automated blood pressure measures (GE DINAMAP 100DPC-120XEN, GE Healthcare) were taken five times each in reclining, sitting and standing after a supine rest for 15 min, and averaged over the 15 assessments to obtain mean systolic blood pressure (BP) and diastolic BP.

### Depressed mood

Depressive symptoms were assessed using the Center for Epidemiological Studies Depression Scale (CES-D) [27] and the Zung self-rating depression scale [28]. The CES-D consists of 20 statements about feelings (e.g., happy, fearful, depressed) and behaviors (e.g., crying, eating, sleeping) and participants are required to indicate how often they have felt or behaved in these ways within the past week on a 4-point scale (from none of the time (0), to all of the time (3)). Total scores range from 0 to 60, with higher scores indicating a greater number of symptoms. A total score of 16 or higher is considered to indicate depressed mood.

The Zung self-rating depression scale [28] consists of 20 statements about feelings and symptoms of depression, such as sleep and appetite. Each statement is rated by the participant on a 4-point scale, ranging from 1) a little of the time, to 4) most of the time. Total scores were multipled by 1.25 to given a final scale ranging from 25 to 100, with higher scores indicating more depressive symptoms. A score of 62.5 or higher is considered to indicate depressed mood. Both scales have demonstrated reliability and validity [29–32]. The use of antidepressant medication was self-reported in the Nutrition and Health Questionnaire [33, 34].

### Other health and demographic variables

Demographic, socioeconomic and lifestyle characteristics were obtained from the Nutrition and Health Questionnaire [33, 34]. Education level (years) was obtained through self-report and ranged from 4 to 20 years. Physical activity was measured with the Nurses’ Health Study Activity Questionnaire, a validated measure of time spent engaging in various physical activities [35]. MET-hours per week for each activity were calculated and summed to give total MET-minutes per week [36]. Smoking status was based on self-report from the Nutrition and Health Questionnaire [37].

Diabetes mellitus was defined as having a fasting plasma glucose ≥ 126 mg/dl or taking anti-diabetic medication, and CVD was considered present if any of the following were present: myocardial infarction, coronary artery disease, heart failure, angina pectoris, or transient ischemic attack.

### Statistical analyses

Participant demographics, health and psychological variables were compared according to MetS status (yes/no). Independent samples *t*-tests were used for continuous variables and Chi-square for categorical variables for the analyses involving demographic variables.

Logistic regression analyses were used to determine whether depressive symptoms (CES-D and Zung) and antidepressant use (yes/no) were associated with MetS. After determining that the relation between depressive symptoms and MetS was not linear, the analysis was performed using scores from each quartile. The three highest quartiles were compared with the lowest quartile (reference group). Odds ratios (OR) and 95 % confidence intervals (CI) were computed. Potential confounding variables were selected if they correlated with both depressive symptoms and MetS, or were of theoretical or clinical importance. The following covariance models were employed to adjust for confounding in the primary analysis:

- Model 1: Basic: age (years) + gender + education (years) + ethnicity;
- Model 2: Basic + smoking (cigarettes/day), physical activity (MET hours/week), CRP (mg/L).

In addition, the odds of having any of the individual MetS components were determined according to depressive symptoms (CES-D and Zung) and antidepressant use in the MSLS. The same two covariate sets were used.

### Sensitivity analyses

Analyses examining relations between depressive symptoms and MetS were performed again, excluding participants reporting use of antidepressants. Further, the same analyses examining potential associations between depressive symptoms and MetS, and between antidepressant use and MetS, were performed after excluding individuals with existing type 2 diabetes or CVD.

To test if any of the potential associations differed for men and women, or according to age, interaction terms (sex*depressive symptoms, sex*antidepressant medication use; age*depressive symptoms, age*antidepressant medication use) were calculated and added to the basic models.

As other studies have shown that the influence of depressive symptoms may be greater for individuals with the apolipoprotein E4 (APOE ε4) allele compared to those without the allele, for example, on cognitive functioning [38], we also tested the possibility that the APOE ε4 allele modifies the relation between depressed mood and MetS, i.e. interaction terms (APOE ε4*depressive symptoms, APOE ε4*antidepressant medication use) were calculated and added to the basic models.

Due to the established effect of depressive symptoms on neuropathologial changes in brain regions, the analyses were repeated with a comprehensive measure of global cognitive function (the MSLS global cognitive composite score) added to the extended model. Finally, as antidepressant medication may impact upon body weight, in a final step, weight was added to the extended model examining the association between antidepressant use and MetS.

## Results

### Demographic and preliminary findings

The demographic, cardiovascular and health variables for MSLS (*N* = 970) participants according to MetS status are shown in Table 1. Those with MetS were slightly older, smoked more, had a higher mean BMI and reported less physical activity. They had significantly lower HDL-, LDL- and total cholesterol than those without MetS; and, as would be expected, they had significantly higher systolic BP, diastolic BP, fasting blood glucose, waist circumference, and triglycerides. Those with MetS reported significantly more depressive symptoms, as measured by both scales, than those without MetS. In total, 110 participants (33 males and 77 females; 11.3 % of the sample), reported using antidepressant medication. Of those taking antidepressants, a significantly higher proportion had MetS than did not. Depressive symptoms were associated with use of antidepressant medication. Those with scores in the highest quartile of depressed mood had significantly higher odds of using antidepressants compared to those with scores in the lowest quartile (basic models: Zung: OR: 4.67, 95 % CI: 2.40–9.08, *P* < 0.001; CES-D: OR: 3.46, 95 % CI: 1.86–6.46, *P* < 0.001) (data not shown). The CES-D and Zung were highly correlated with each other (Pearsons *r* = 0.61, *P* < 0.001).

### Main findings

#### Depressive symptoms and MetS

Those with the highest quartile of scores on the CES-D had a 79 % higher risk of having MetS (basic model, OR: 1.79, *P* < 0.01), and those with the highest quartile of scores on the Zung had a 71 % higher risk of having MetS (basic model, OR: 1.71, *P* < 0.01), compared to those with scores in the lowest quartile (Table 2). Those with scores in the second and third quartiles did not differ significantly (*P* > 0.05) from those with the lowest scores in terms of MetS risk, for either of the depressed mood measures.

**Table 2:** Odds of having metabolic syndrome according to presence of depressive symptoms and use of antidepressant medications in the Maine-Syracuse Longitudinal Study

Depressive symptoms/medication use	Covariate set^a^	Quartile of scores^b^	MetS		
			OR	95 % CI	*P*-value
CES-D score	Basic	Q4	1.79	1.22, 2.62	0.003
		Q3	1.35	0.93, 1.97	0.1
		Q2	1.05	0.72, 1.54	0.8
		Q1	1.00		
	Extended	Q4	1.50	1.00, 2.24	0.049
		Q3	1.23	0.83, 1.82	0.3
		Q2	0.99	0.66, 1.49	0.9
		Q1	1.00		
Zung score	Basic	Q4	1.71	1.17, 2.50	0.006
		Q3	1.20	0.81, 1.77	0.4
		Q2	1.14	0.79, 1.65	0.5
		Q1	1.00		
	Extended	Q4	1.43	0.99, 2.50	0.1
		Q3	0.99	0.66, 1.52	0.9
		Q2	1.05	0.71, 1.54	0.8
		Q1	1.00		
Antidepressant medication use	Basic	Yes	2.31	1.51, 3.54	<0.001
		No	1.00		
	Extended	Yes	2.22	1.42, 3.48	<0.001
		No	1.00		

*CES-D* Center for Epidemiologic Studies Depression Scale, *CI* confidence interval, *MetS* metabolic syndrome, *OR* odds ratio

^a^Basic: adjusted for age, education, sex, ethnicity; Extended: adjusted for set 1 + smoking (cigarettes per day), physical activity (MET-minutes/day), CRP (mg/L)

^b^Quartile 1 (lowest quartile of scores) = reference group

For the extended model (addition of risk factor covariates), relations between depressed mood and MetS seen with the basic mode (age, education, gener and ethnicity) remained significant for the CES-D, with some attenuation of risk (see Table 2). Those with scores in the highest qurtile on the CES-D had a significantly higher risk for MetS, compared to those with scores in the lowest quartile.

### Antidepressant medication use and MetS

Those on antidepressants (*n* = 110, 11.3 % of the sample) had slightly over a two-fold increase in relative risk for MetS, compared to those not taking antidepressants (extended model: OR = 2.22, *P* < 0.001) (Table 2).

### Depressive symptoms, antidepressant medication and individual MetS components

Those with the highest scores on both the CES-D and the Zung (highest quartiles) had a significantly increased likelihood of having low HDL-cholesterol (CES-D: OR: 1.89, *P* < 0.01; Zung: OR: 1.48, *P* < 0.05; basic model) (Table 3). The association between higher CES-D scores and risk for low HDL-cholesterol remained significant with addition of covariates, but was no longer significant for the Zung. Neither depressed mood measure was associated with any of the other individual MetS components.

**Table 3:** Odds of having individual metabolic syndrome components according to depressed mood in the Maine-Syracuse Longitudinal Study

Depressive symptoms	Covariate set^a^	Quartile of scores^b^	MetS low HDL-cholesterol^c^		
			OR	95 % CI	*P*
CES-D score	Basic	Q4	1.89	1.30, 2.74	0.001
		Q3	1.28	0.89, 1.85	0.2
		Q2	1.41	0.98, 2.04	0.1
		Q1	1.00		
	Extended	Q4	1.67	1.13, 2.46	0.010
		Q3	1.21	0.83, 1.77	0.3
		Q2	1.34	0.91, 1.98	0.1
		Q1	1.00		
Zung score	Basic	Q4	1.48	1.02, 2.14	0.037
		Q3	1.36	0.93, 1.99	0.1
		Q2	1.01	0.71, 1.44	0.9
		Q1	1.00		
	Extended	Q4	1.30	0.88, 1.91	0.2
		Q3	1.26	0.84, 1.87	0.3
		Q2	0.99	0.69, 1.44	0.9
		Q1	1.00		

*CES-D* Center for Epidemiologic Studies Depression Scale; *CI* confidence interval; *MetS* metabolic syndrome; *OR* odds ratio

^a^Basic: adjusted for age, education, sex, ethnicity; Extended: adjusted for set 1 + smoking (cigarettes per day), physical activity (MET-minutes/day), CRP (mg/L)

^b^Quartile 1 (lowest quartile of scores) = reference group

^c^MetS clinical cut off points: reduced HDL-cholesterol: <1.0 mmol/L or <40 mg/dL for males; <1.3 mmol/L or <50 mg/dL for females [1]

Antidepressant use was significantly associated with higher odds for having elevated glucose levels, hypertension, and low HDL-cholesterol with full covariate adjustment (extended models, all *P* < 0.05) (Table 4).

**Table 4:** Odds of having individual metabolic syndrome components according to use of antidepressant medications in the Maine-Syracuse Longitudinal Study

Predictor	Covariate set^a^	Group	MetS elevated glucose^b^			MetS hypertension^b^			MetS low HDL-cholesterol^b^		
			OR	95 % CI	*P*	OR	95 % CI	*P*	OR	95 % CI	*P*
Antidepressant medication use	Basic	Yes	2.01	1.30, 3.13	0.002	1.99	1.21, 3.25	0.006	1.66	1.10, 2.51	0.015
		No	1.00			1.00			1.00		
	Extended	Yes	1.95	1.23, 3.09	0.005	1.81	1.09, 3.02	0.023	1.57	1.03, 2.40	0.036
		No	1.00			1.00			1.00		

CI, confidence interval; MetS, metabolic syndrome; OR, odds ratio

^a^Basic: adjusted for age, education, sex, ethnicity; Extended: adjusted for set 1 + smoking (cigarettes per day), physical activity (MET-minutes/day), CRP (mg/L)

^b^MetS clinical cut off points: elevated fasting plasma glucose: ≥5.6 mmol/L or ≥100 mg/dL; elevated blood pressure: systolic: ≥130 and/or diastolic ≥85 mmHg; reduced HDL-cholesterol: <1.0 mmol/L or <40 mg/dL for males; <1.3 mmol/L or <50 mg/dL for females [1]

### Secondary analyses

In a secondary set of analyses, neither of the age, sex or APOE ε4 interaction terms added to the basic model were significant (all *P* values >0.4, data not shown). Excluding participants with either CVD or diabetes did not change the pattern of results. Controlling for cognition (global composite score) in the extended model did not change the results. Finally, there was no attenuation of the results when body weight was added to the extended model testing the association between antidepressant use and MetS.

### Availability of data and materials

The dataset supporting the conclusions of this article are available from the authors upon request.

## Discussion

We have observed a significant association between elevated depressive symptoms and greater prevalence of MetS, controlling for socio-demographics, lifestyle factors and CRP. The findings were consistent across two different scales measuring the same construct (basic model). Few, if any, studies have examined relations between depressed mood using two different scales and this is necessary to the development of consistent operational definitions of depressed mood. Good cognitive studies, as an example, employ more than one index of a cognitive domain to define that domain. Our second major finding was that antidepressant use was associated with over 2-fold increased risk for MetS, adjusted for socio-demographics, lifestyle factors and CRP. Of the individual MetS components, low HDL-cholesterol was related to both measures of depressed mood, and antidepressant use was significantly related to elevated glucose levels, hypertension, and low HDL-cholesterol.

The results of the analyses indicate that the risk for MetS is not distributed equally across all quartiles of the depressed mood scale distribution. It was the individuals with depressed mood scores in the highest quartile that exhibited a significantly increased risk for MetS, compared to those individuals with scores in the lowest quartile. This makes sense from a clinical perspective as one would expect that the more extreme levels of depressed mood would be related to MetS. In the extended model, these statistically significant associations between the highest quartile of scores and MetS were attenuated but remained significant for the CES-D. This suggests that the presence of cardiovascular risk factors, such as inflammation, smoking or physical activity, attenuate the relations between depressed mood and MetS, and that this phenomenon is related to the fact that depressed mood raises the risk for CVD [8] and conversely the presence of CVD can lead to depression [39].

As a distinguishing factor from other studies, we were able to also assess if the results varied according to APOE ε4 status. No difference was found between those with and without at least one allele. The study included participants across a wide age range. Age may impact upon one’s cognitive abilities in relation to self-reported depressive symptoms. We found no evidence for an interaction between age and the depressive symptom measures. In addition, statistically controlling for cognitive function (adding the global composite score to the extended model) did not change the results.

Our findings are consistent with numerous other studies which have shown positive associations between depression and MetS. Pan et al [10] reviewed both cross-sectional studies, and prospective studies that reported odds ratios for depression as the outcome, and OR for MetS as the outcome. Depression and MetS were correlated in cross-sectional studies, and a bidirectional association was observed in the prospective studies. Multivariate logistic regression with depression as the dependent variable yielded an OR of 1.27 (95 % CI 1.07–1.51) (based on 11 studies). Of note, this review included studies using both a self-reported symptom scale and diagnostic interview to assess depression. Interestingly, the association was slightly weaker when depression was assessed by diagnostic interview rather than self-reported (OR = 1.29 vs. 1.51) [10]. Data on antidepressant medication use was not examined in this review.

Our study examined depressive symptoms, assessed through the completion of self-rated scales, reliant upon the participant’s perception of symptom burden. Other studies have demonstrated a similar pattern of association with clinically diagnosed depressive disorders established objectively using standardized diagnostic interviews. A number of studies have shown positive relations between major depressive disorder (both current and lifetime) and MetS [11–13]. For example, Butnoriene et al [11] showed a significant relation between major depressive disorder (both current and lifetime) and depressive symptoms with MetS prevalence in a community sample. Data from the National Health and Nutrition Examination Survey (NHANES III) obtained from young adults found that a history of major depression was associated with increased odds of MetS in women, but not in men [13]. Similarly, Goldbacher et al [40] showed that a lifetime history of major depression predicted an increased risk of developing MetS in middle-aged women.

We found that depressive symptoms may be directly associated with HDL-cholesterol levels. Other studies investigating depression in relation to the individual components of MetS have shown significant and independent associations between low HDL-cholesterol [20, 41], as well as large waist circumference [20, 41], hypertension [13], and high triglycerides [13, 20] with depression. In the present study, antidepressant use was associated with higher glucose and blood pressure levels, and lower HDL-cholesterol. Use of antidepressant medication has been previously associated with higher triglycerides, systolic BP, and waist circumference [20]. Weight gain during antidepressant treatment can be either a sign of improvement in patients who have weight loss as a symptom of depression, or may be a residual symptom of weight gain from overeating when depressed [18]. Substantial weight gain during the early phase of treatment with antidepressants or weight gain that continues despite the resolution of depressive symptoms may be a side-effect of the drug treatment [18]. A review by Newcomer and Haupt [42] indeed concluded that antipsychotic medications may be associated with adverse metabolic effects, including weight gain and decreases in insulin sensitivity, which may further contribute to increasing plasma glucose and lipid levels, and therefore MetS risk.

### Mechanisms

There are a number of potential mechanisms that may underlie the association between depressed mood and MetS. Firstly, depression has been associated with obesity [43], inflammation [44], and insulin resistance [45], which are all linked with MetS. Inflammation as a consequence of obesity is likely to be a central component [41, 46]. Depression may facilitate physiological changes, such as dysregulation of the hypothalamus-pituitary-adrenal (HPA) axis, resulting in increased secretion of corticotrophin-releasing hormone, adrenocorticotropic hormone, and cortisol [47]. This promotes deposition of visceral fat tissue, which secretes inflammatory cytokines implicated in insulin resistance, a key factor in MetS. Cortisol also inhibits lipid mobilization, directly effecting dyslipidemia [14, 41].

Alternatively, depressed mood may result in increased consumption of food and/or alcohol as a means for coping with negative emotion, promoting weight gain and subsequently activating an inflammatory response [12]. Other negative, depression-related behaviours, such as the consumption of an unhealthy diet, engaging in little physical activity and poor sleep patterns may enhance oxidative stress, adiposity, inflammation, and an increased risk for metabolic disturbances [12, 48, 49].

### Implications

Individuals with elevated depressive symptoms, even without major depressive disorder, and individuals taking antidepressant medication should be considered at risk for MetS. Assessment and monitoring of MetS components should be routine care for these people [50]. Lifestyle changes could be recommended if patients are found to be at increased risk for cardiometabolic risk factors. The use of self-reported depression symptom scales are therefore clinically important for identifying individuals at risk for more severe depression and metabolic sequela. Contrary to a few studies and consistent with others we find no relation higher risk for MetS in persons carrying one or two APOE ε4 alleles.

### Limitations

Due to the cross-sectional design, we are only able to infer an association between elevated depressive symptoms, antidepressant use, and higher MetS prevalence. No inferences regarding causality can be made. The results may not be generalizable to the African American community as they only comprised a small proportion of the sample (7.5 %). Finally, not all possible relevant lifestyle behaviors or social psychological variables were included in the study. Other inflammatory markers, such as leptin and adiponectin were not measured. We also did not have data on family history of depression or disease, which may have an effect on our findings, but was not a question raised in this study. Absence of data on duration of treatment with antidepressant medications is a limitation, albeit our major outcomes for inferring depression (more specifically depressed mood) were the two depressed mood scales.

### Strengths

The major strengths of the study include the representative, community-based sample of men and women, the assessment of MetS criteria, depressive symptoms, cardiovascular risk varibles and other health and lifestyle factors, and the fact that examiners were blind to the subject’s MetS status.

## Conclusions

Depressive symptoms are associated with an increased prevalence of MetS. Those taking antidepressants have over twice the likelihood of having MetS, compared with those not taking such medication. Well-designed longitudinal studies are required to examine the chronological sequence of the development of depression and MetS. More studies are also needed to examine the biological and behavioral mechanisms underlying this depression-MetS relation, important for the prevention and management of both.

## Abbreviations

APOE ε4, apolipoprotein ε4; BMI, body mass index; BP, blood pressure; CES-D, Center for Epidemiological Studies Depression Scale; CI, confidence interval; CRP,C-reactive protein; CVD, Cardiovascular disease; HDL, High-density lipoprotein; LDL, Low-density lipoprotein; MET, metabolic equivalent; MetS, Metabolic Syndrome; MSLS, Maine-Syracuse Longitudinal Study; OR, odds ratio; TC, total cholesterol
